# Cardiovascular Function in Pulmonary Emphysema

**DOI:** 10.1155/2013/184678

**Published:** 2013-12-03

**Authors:** Dina Visca, Marina Aiello, Alfredo Chetta

**Affiliations:** Respiratory Disease & Lung Function Unit, Department of Clinical & Experimental Medicine, University of Parma, Padiglione Rasori, Via G. Rasori 10, 43125 Parma, Italy

## Abstract

Chronic obstructive pulmonary disease (COPD) and chronic cardiovascular disease, such as coronary artery disease, congestive heart failure, and cardiac arrhythmias, have a strong influence on each other, and systemic inflammation has been considered as the main linkage between them. On the other hand, airflow limitation may markedly affect lung mechanics in terms of static and dynamic hyperinflation, especially in pulmonary emphysema, and they can in turn influence cardiac performance as well. Skeletal mass depletion, which is a common feature in COPD especially in pulmonary emphysema patients, may have also a role in cardiovascular function of these patients, irrespective of lung damage. We reviewed the emerging evidence that highlights the role of lung mechanics and muscle mass impairment on ventricular volumes, stroke volume, and stroke work at rest and on exercise in the presence of pulmonary emphysema. Patients with emphysema may differ among COPD population even in terms of cardiovascular function.

## 1. Introduction

Pulmonary emphysema, a phenotype of chronic obstructive pulmonary disease (COPD), is a pathologic condition characterized by abnormal and permanent enlargement of the airspaces distal to the terminal bronchioles that leads to airspace walls destruction and usually to progressive airflow limitation [1]. In pulmonary emphysema, the loss of elastic recoil leads not only to the irreversible bronchial obstruction, but also to the lung hyperinflation, which implies an increased volume over the normal tidal breathing range and an increase in functional residual capacity (FRC). Furthermore, the more lung function is impaired, the more the airway collapsibility affects lung mechanics, leading to a high intrinsic positive end-expiratory pressure (PEEPi) that increases intrapleural pressure.

The emphysema phenotype defines COPD patients, who complain of dyspnoea and reduced exercise capacity, as predominating symptoms. Skeletal muscle depletion and malnutrition may also characterize emphysema patients. An inverse correlation was found between body mass index (BMI) and degree of emphysema, evaluated by high-resolution computed tomography [2].

There is an increasing body of evidence that, in COPD patients, a chronic cardiovascular disorder, such as coronary artery disease or congestive heart failure, may be a frequent comorbidity because of smoking habit, which is a common risk factor, and that the inflammation associated with COPD is not limited to the lung, but it can also affect nonpulmonary organs, such as the cardiovascular system [3–5]. Interestingly, in pulmonary emphysema changes both in lung mechanics and in skeletal muscle pump may impair *per se* the cardiovascular function. This overview, therefore, is specifically addressed to the cardiovascular system function in patients with pulmonary emphysema.

## 2. Heart and Pulmonary Hyperinflation

Pulmonary hyperinflation can significantly affect heart size and its function. By means of magnetic resonance technique, Jörgensen et al. [6] studied patients with severe emphysema and found a decrease in intrathoracic blood volume and in left ventricular and right ventricular end-diastolic volumes and an impaired stroke volume and stroke work in hyperinflated lungs, as compared to controls. The authors argued that there are at least two main explanations of these findings. In presence of hyperinflated lungs, a high PEEPi could cause intrathoracic hypovolemia and small end-diastolic dimensions of both left and right ventricular chambers. The redistribution of pulmonary circulation in emphysema might occur not only because of a direct parenchymal destruction or hypoxia vasoconstriction, but also because of a decreased compliance of pulmonary vascular bed that tends to push blood to the periphery borne down by a high PEEPi. Secondly, right and left ventricular chambers could be mechanically compressed by hyperinflated lungs that could worsen end-diastolic stiffness. According to the Frank-Starling law, a low preload finally reduces ventricular performance in terms of stroke volume (SV) and stroke work.

In a large sample of COPD patients, ranging from GOLD I to IV class, Watz et al. [7] found that the degree of COPD severity was directly correlated to heart dysfunction. Interestingly, in this study, the cardiac chamber sizes and impaired left ventricular diastolic filling pattern correlated more to the degree of static hyperinflation, as assessed by inspiratory capacity-to-total lung capacity ratio (IC/TLC), than to the degree of airway obstruction, expressed as forced expiratory volume in 1st second (FEV_1_) % predicted, or to diffusion capacity to carbon monoxide. Furthermore, IC/TLC was an independent predictor of cardiac chamber sizes after adjustment for body surface area [7].

In line with the findings by Watz et al. [7], Malerba et al. [8] reported a frequent subclinical left ventricular filling impairment in COPD patients at the earlier stage of the disease, even in the absence of any other cardiovascular disorder. Furthermore, Smith et al. [9] have recently shown a reduction of pulmonary vein dimension in COPD, which is related to percent emphysema, thereby supporting a mechanism of upstream pulmonary causes for left ventricle underfilling.

Interestingly, as the pulmonary hyperinflation may have negative effects, so the pulmonary deflation has the potential to improve the cardiac function in patients with pulmonary emphysema. In patients severely hyperinflated, Come and coworkers [10] have recently found that the decreased hyperinflation through lung volume reduction surgery (LVRS) was significantly associated with an improvement in oxygen pulse, which may be considered as a noninvasive marker of cardiovascular efficiency and a measure of SV.

It is of note that the extent of emphysema, as detected on computed tomography (CT), may be associated with an impaired cardiac function, even in patients without very severe lung disease [11]. In a recent population-based study, a greater extent of emphysema on CT scanning was linearly related to impaired left ventricular filling, reduced stroke volume, and lower cardiac output without changes in the ejection fraction [11]. The smoking status significantly worsened these associations. Accordingly, the authors [11] hypothesized that the mechanisms of the impaired left ventricular filling in early, mild emphysema might be the subclinical loss of capillary bed due to the apoptotic effect of smoking on pulmonary endothelium.

## 3. Cardiovascular Response to Exercise and Dynamic Hyperinflation

In healthy subjects at rest, FRC physiologically equals the relaxation volume (*V*_*r*_), at which all respiratory muscles are relaxed and the outward elastic recoil of the chest wall precisely balances the inward recoil of the lungs. By contrast, in patients with expiratory flow limitation, changes in ventilation, such as an increase in flow and/or in breathing frequency, can elevate FRC above *V*_*r*_. The condition characterized by FRC which is not equal to but greater than *V*_*r*_  is called “dynamic hyperinflation” and may typically occur during exercise in COPD patients. In addition to the static lung hyperinflation, dynamic hyperinflation is responsible for limitation to exercise in COPD patients and for onset of exertion dyspnoea [12]. Accordingly, it is conceivable that during exercise dynamic hyperinflation can further worsen a poor resting cardiac function in patients with pulmonary emphysema.

Both ventilatory and cardiac responses to exercise can be well studied through cardiopulmonary exercise test (CPET). CPET is a relatively noninvasive method to test tolerance to maximal exercise and gives several pieces of information about how cardiovascular, respiratory, and muscle apparatuses respond to exercise. Notably, the assessment of the dynamic hyperinflation is based on the comparison of the IC performed at rest and during exercise and a positive difference between them is putative of dynamic hyperinflation, assuming that TLC remains constant during exercise.

Dynamic hyperinflation, which impairs the cardiovascular function in COPD patients, may be documented during rapidly incremental CPET. Vassaux et al. [13] first observed that dynamic hyperinflation is negatively associated with oxygen pulse at peak of exercise in patients with severe COPD. These results were confirmed and extended by our group in COPD patients with different degrees of severity, by showing a significant relationship between dynamic hyperinflation and a battery of noninvasive measures of cardiovascular function during exercise [14]. Notably, in these patients, we found that the extent of dynamic hyperinflation was inversely related not only to oxygen pulse, but also to the product of systolic blood pressure and heart rate, the so-called double product (DP). Interestingly, DP reflects myocardial oxygen uptake during exercise because the three major determinants of myocardial oxygen uptake are the tension in the wall of the ventricle, the contractile state of the heart, and the heart rate [15]. Secondly, we observed that the oxygen uptake efficiency slope (OUES), a parameter that integrates the functional capacities of several organ systems (cardiovascular, musculoskeletal, and pulmonary) and that represents the rate of increased O_2_ consumption in response to a given ventilation during incremental exercise [16], was negatively associated with dynamic hyperinflation.

Importantly, it has been recently shown that in patients with severe pulmonary emphysema the reduction in dynamic hyperinflation after LVRS was significantly associated with an improvement in cardiac response to exercise, both in terms of oxygen pulse and pulse pressure, which is the difference between systolic and diastolic blood pressure [17]. It is of note that pulmonary rehabilitation may lower the ventilatory demand during exercise, resulting in the prolongation of the expiration time and, in turn, in the reduction of dynamic hyperinflation [18]. Accordingly, one may hypothesize that, in COPD patients, pulmonary rehabilitation may improve the cardiovascular response to exercise by enhancing the ventilatory function. In line with this assumption, our group has recently reported an improvement in cardiovascular response during exercise at submaximal exercise independent of the external work after a standard pulmonary rehabilitation program [19]. This change was significantly associated with an enhancement in ventilatory function during exercise.

## 4. Skeletal Muscle Pump and Cardiovascular Function

Skeletal muscle pump is of basic importance both in local and systemic circulatory effects, since it may enhance venous return, central venous pressure, end-diastolic volume, and thus stoke volume and cardiac output, by expulsing the peripheral venous blood volume during exercise [20]. In this way, the muscle pump makes also more blood flow available to be diverted to active muscle and thereby indirectly inducing muscle hyperemia [20]. On the other hand, a skeletal muscle depletion may negatively affect the cardiovascular response to exercise.

In COPD patients, a skeletal muscle depletion may commonly occur, resulting from several factors, such as disuse atrophy, poor nutrition, systemic inflammatory mediators, and oral corticosteroids chronically administered [21]. Importantly, when in a COPD population the characterization of phenotypes was based on the presence and the severity of emphysema, patients with the phenotype in which emphysema predominates have significantly lower BMI [22].

Recently, our group has shown that the muscle mass depletion plays a part *per se* in the reduction of exercise capacity of COPD patients, regardless of lung function impairment, and is strictly associated with poor cardiovascular response to exercise and to leg fatigue [23]. Notably, in our study, both resting and peak oxygen pulse values were significantly lower in depleted patients, as compared to nondepleted, whereas peak oxygen pulse value was strongly related to fat-free mass in the whole population. We also found that depleted and nondepleted patients differed in OUES, which is an objective measure of cardiorespiratory and muscular fitness [24]. Lastly, in our patients, we found that the heart rate recovery after a maximal exercise, a marker of the cardiac autonomic function and a powerful predictor of mortality in the general population [25], was significantly lower in depleted patients.

## 5. Conclusions

There is growing recognition that COPD and chronic cardiac diseases appear to be linked by an underlying systemic inflammatory status. At the same time, lung mechanics and cardiac performance are deeply dependent on each other and both may be responsible for exercise limitation, exertion dyspnoea, and poor quality of life in the presence of irreversible airflow limitation and lung hyperinflation. Furthermore, muscle mass depletion, which especially characterizes patients with pulmonary emphysema among COPD population, may also contribute to cardiovascular response to exercise.

Clinicians should take into consideration that any therapeutic approach, such as inhaled bronchodilators, lung volume reduction surgery, and pulmonary rehabilitation, that aims to improve lung mechanics may in turn improve cardiac performance as well in COPD patients. Furthermore, all that can increase muscle mass in depleted COPD patients may in turn improve their cardiovascular function.

In conclusion, emphysema and chronic bronchitis are two different phenotypes of COPD not only from a clinical and functional point of view, but also in terms of cardiovascular function. Notably, systemic inflammation, lung mechanics impairment, and muscle mass depletion might play a different role in conditioning cardiovascular function in patients with emphysema and in patients with chronic bronchitis. Further studies are requested to address this matter and to provide solutions.

## Abbreviations

BMI: Body mass index
COPD: Chronic obstructive pulmonary disease
CPET: Cardiopulmonary exercise test
CT: Computed tomography
DP: Double product
FEV_1_: Forced expiratory volume in 1st second
GOLD: Global initiative for chronic obstructive lung disease
IC: Inspiratory capacity
LVRS: Lung volume reduction surgery
OUES: Oxygen uptake efficiency slope
PEEPi: Intrinsic positive end-expiratory pressure
SV: Stroke volume
TLC: Total lung capacity.
